# GWAS and enrichment analyses of non-alcoholic fatty liver disease identify new trait-associated genes and pathways across eMERGE Network

**DOI:** 10.1186/s12916-019-1364-z

**Published:** 2019-07-17

**Authors:** Bahram Namjou, Todd Lingren, Yongbo Huang, Sreeja Parameswaran, Beth L. Cobb, Ian B. Stanaway, John J. Connolly, Frank D. Mentch, Barbara Benoit, Xinnan Niu, Wei-Qi Wei, Robert J. Carroll, Jennifer A. Pacheco, Isaac T. W. Harley, Senad Divanovic, David S. Carrell, Eric B. Larson, David J. Carey, Shefali Verma, Marylyn D. Ritchie, Ali G. Gharavi, Shawn Murphy, Marc S. Williams, David R. Crosslin, Gail P. Jarvik, Iftikhar J. Kullo, Hakon Hakonarson, Rongling Li, The eMERGE Network, Stavra A. Xanthakos, John B. Harley

**Affiliations:** 1 Center for Autoimmune Genomics and Etiology, Cincinnati Children’s Hospital Medical Center (CCHMC), Cincinnati, OH USA; 2 College of Medicine, University of Cincinnati, 3333 Burnet Avenue, Cincinnati, OH 45229 USA; 3 Division of Biomedical Informatics, Cincinnati Children’s Hospital Medical Center, Cincinnati, OH USA; 4 Department of Biomedical Informatics Medical Education, School of Medicine, University of Washington, Seattle, WA USA; 5 Center for Applied Genomics, Children’s Hospital of Philadelphia, Bethesda, MD USA; 6 Research IS and Computing, Partners HealthCare, Harvard University, Somerville, MA USA; 7 Departments of Biomedical Informatics and Medicine, Vanderbilt University, Nashville, TN USA; 8 Center for Genetic Medicine, Northwestern University Feinberg School of Medicine, Chicago, IL USA; 9 Division of Immunobiology, Department of Pediatrics, Cincinnati Children’s Hospital Research Foundation and the University of Cincinnati College of Medicine, Cincinnati, OH USA; 10 Kaiser Permanente Washington Health Research Institute (Formerly Group Health Cooperative-Seattle), Kaiser Permanente, Seattle, WA USA; 11 Department of Molecular and Functional Genomics, Geisinger, Danville, PA USA; 12 Department of Genetics, University of Pennsylvania, Philadelphia, PA USA; 13 Department of Medicine, Columbia University, New York City, NY USA; 14 Research Information Science and Computing, Partners HealthCare, Boston, MA USA; 15 Genomic Medicine Institute (M.S.W.), Geisinger, Danville, PA USA; 16 Departments of Medicine (Medical Genetics) and Genome Sciences, University of Washington Medical Center, Seattle, WA USA; 17 Department of Cardiovascular Diseases, Mayo Clinic, Rochester, MN USA; 18 Perelman School of Medicine at the University of Pennsylvania, Philadelphia, PA USA; 19 National Human Genome Research Institute, National Institutes of Health, Bethesda, MD USA; 20 Division of Gastroenterology, Hepatology and Nutrition, Department of Pediatrics, Cincinnati Children’s Hospital Medical Center, University of Cincinnati School of Medicine, Cincinnati, OH USA; 21 U.S. Department of Veterans Affairs Medical Center, Cincinnati, OH USA

**Keywords:** NAFLD, Fatty liver, Genetic polymorphism, GWAS, PheWAS, Polygenic risk score

## Abstract

**Background:**

Non-alcoholic fatty liver disease (NAFLD) is a common chronic liver illness with a genetically heterogeneous background that can be accompanied by considerable morbidity and attendant health care costs. The pathogenesis and progression of NAFLD is complex with many unanswered questions. We conducted genome-wide association studies (GWASs) using both adult and pediatric participants from the Electronic Medical Records and Genomics (eMERGE) Network to identify novel genetic contributors to this condition.

**Methods:**

First, a natural language processing (NLP) algorithm was developed, tested, and deployed at each site to identify 1106 NAFLD cases and 8571 controls and histological data from liver tissue in 235 available participants. These include 1242 pediatric participants (396 cases, 846 controls). The algorithm included billing codes, text queries, laboratory values, and medication records. Next, GWASs were performed on NAFLD cases and controls and case-only analyses using histologic scores and liver function tests adjusting for age, sex, site, ancestry, PC, and body mass index (BMI).

**Results:**

Consistent with previous results, a robust association was detected for the PNPLA3 gene cluster in participants with European ancestry. At the PNPLA3-SAMM50 region, three SNPs, rs738409, rs738408, and rs3747207, showed strongest association (best SNP rs738409 *p* = 1.70 × 10^− 20^). This effect was consistent in both pediatric (*p* = 9.92 × 10^− 6^) and adult (*p* = 9.73 × 10^− 15^) cohorts. Additionally, this variant was also associated with disease severity and NAFLD Activity Score (NAS) (*p* = 3.94 × 10^− 8^, beta = 0.85). PheWAS analysis link this locus to a spectrum of liver diseases beyond NAFLD with a novel negative correlation with gout (*p* = 1.09 × 10^− 4^). We also identified novel loci for NAFLD disease severity, including one novel locus for NAS score near *IL17RA* (rs5748926, *p* = 3.80 × 10^− 8^), and another near *ZFP90-CDH1* for fibrosis (rs698718, *p* = 2.74 × 10^− 11^). Post-GWAS and gene-based analyses identified more than 300 genes that were used for functional and pathway enrichment analyses.

**Conclusions:**

In summary, this study demonstrates clear confirmation of a previously described NAFLD risk locus and several novel associations. Further collaborative studies including an ethnically diverse population with well-characterized liver histologic features of NAFLD are needed to further validate the novel findings.

**Electronic supplementary material:**

The online version of this article (10.1186/s12916-019-1364-z) contains supplementary material, which is available to authorized users.

## Background

Nonalcoholic fatty liver disease (NAFLD) is one of the most common chronic liver diseases, found in 17–30% of the population in Western countries [1]. NAFLD, defined as greater than 5% fatty acid content of liver by weight, includes not only simple and benign steatosis but also the more serious nonalcoholic steatohepatitis (NASH), which may progress to cirrhosis and liver failure in 8 to 26% of adults with NASH [2]. NASH is defined histologically by the presence of macrovesicular steatosis, lobular inflammation, and hepatocellular ballooning. The pathology is often indistinguishable from alcoholic fatty liver disease; therefore, the diagnosis can only be made in the absence of significant alcohol use [3]. NAFLD is now recognized as a common metabolic disorder globally as a result of ongoing obesity pandemic. It also increases risk of adverse long-term consequences including death from liver cirrhosis and cardiovascular disease. In fact, NASH is now the second most common indication for liver transplantation in the USA after chronic hepatitis C [4].

Growing evidence has shown that NAFLD can also occur in 10–20% of non-obese population, most often in association with central adiposity, recent weight gain, dietary factors, or genetic risk alleles [5]. In East Asian countries, for example, the incidence and prevalence of NAFLD are increasing with time despite lower rates of obesity compared to Western countries [6]. Hence, it is important to identify the natural course of NAFLD and the contributing factors for the development and maintenance or regression of this disease. The underlying etiology is believed to be multifactorial with a substantial genetic component. The heritability estimates of NAFLD generally range from 20 to 70%, depending on the study design, ethnicity, and the methodology used [7]. Likewise, for indices of disease severity, the heritability estimates in a twin study for hepatic steatosis was 0.52 (based on MRI proton-density fat fraction) and for liver fibrosis (based on liver stiffness) 0.5 [8]. In addition, heritability risk for NAFLD may be independent of body mass index heritability. For example, family studies show that while fatty liver can be present in 17% of siblings and 37% of parents of overweight children without NAFLD, it was significantly more common in siblings (59%) and parents (78%) of children with NAFLD [9]. To date, several genome-wide association studies (GWAS) have been published for this condition mainly in adult cohorts [10–12]. One of the established effects is in the *PNPLA3* (patatin-like phospholipase domain–containing 3) gene with consistent results across studies in which the rs738409 C>G variant (resulting in an amino acid substitution of methionine for isoleucine at position 148 (I148M)) is strongly associated with this trait. The PNPLA3 protein exerts lipase activity and plays a role in the hydrolysis of glycerolipids, with maximum enzymatic activity against triglycerides, diacylglycerol, and monacylglycerol [13]. Structural modeling suggests that this substitution may occlude access of substrates to the catalytic dyad [14]. However, the exact underlying mechanisms remain unclear.

The electronic medical record (EMR) is a rich source of clinical information. Natural language processing (NLP) techniques have demonstrated successes within the clinical domain and have been tested for transferability to another institution [15]. The electronic MEdical Records and GEnomics (eMERGE) Network, founded in 2007, is a consortium of multiple adult and pediatric institutions developed to explore the utility of DNA biorepositories linked to EMRs as well as establishing and validating specific algorithms with and without NLP for many common phenotypes [16]. In this study, we investigated the genetic variants associated with NAFLD/NASH in children and adults using phenotypic measures extracted from medical records in a collection of already genotyped samples from more than 80,000 eMERGE participants to replicate prior studies and identify additional genetic loci.

## Methods

### Study participants and phenotype

Data for this study were collected from the eMERGE Network [17]. Protocols for this study were approved by the Institutional Review Boards (IRBs) at the institutions where participants were recruited; all included participants provided written informed consent prior for inclusion in the study. The population comprised 9677 unrelated European ancestry participants (1106 cases and 8571 controls). A natural language processing (NLP) algorithm was deployed in each site to identify NAFLD cases and controls. These include logic concepts using billing codes, laboratory values, text queries, and medication records to identify true cases and controls at each site. A rules-based NLP algorithm was developed using structured and unstructured data from Cincinnati Children’s Hospital and Medical Center (CCHMC) and secondarily validated in Children’s Hospital of Philadelphia (CHOP) with high precision. The eMERGE protocol includes development of an algorithm at a primary site and the implementation and validation at a secondary site. The secondary site serves as a testing ground for the purposes of mitigating overfitting concerns and ensuring portability. Expert validation includes manual chart review at each site by a physician for both cases and controls. After obtaining a validated positive predictive value of 95% for cases and controls at both the primary (CCHMC) and secondary sites (CHOP), the algorithm has been implemented across network. The exclusion and inclusion criteria for NAFLD were derived according to recommendation from the American Association for the Study of Liver Diseases (AASLD) practical guideline for NAFLD [18]. Case inclusion and exclusion criteria, list of excluded medications, and the number of participants per eMERGE site can be found in Additional file 1: Table S1. We processed the pathology and radiology reports from encounters with diagnosis codes by searching with regular expressions for specific related terms as shown in Additional file 1: Table S1. The NegEx multilingual lexicon was used to assess positive and negative condition for each term [19]. In addition, NAFLD disease severity was assessed based on available liver enzyme and histopathologic grade using the NAFLD Activity Score (NAS). NAS score is a standard method used to score NAFLD disease activity and originally has been developed as a tool to measure disease prognosis and changes in NAFLD during therapeutic trials [20]. The NAS is derived from an unweighted sum of scores of liver steatosis (0–3), lobular inflammation (0–3), and hepatocellular ballooning (0–2), ranging between 0 and 8. Coexistent fibrosis also has a separate scoring range of 0–4. This consists of no fibrosis (0), perisinusoidal or periportal (1), portal (2), bridging fibrosis (3), and cirrhosis (4). The NAS classification scoring system is shown in Additional file 1: Table S1. We obtained these values from pathology reports using NLP processing for 235 of our NAFLD case participants. In addition, for each case, the highest level of liver enzyme values for aspartate aminotransferase (AST U/L) and alanine aminotransferase (ALT U/L) was obtained for association testing.

### Genotyping and imputation

Genetic data for the eMERGE Network is available from the coordinating center and can be accessed through dbGAP (phs000888.v1.p1) which is annually updated. High-throughput SNP genotyping was carried out previously in each contributing medical center. A series of standard quality control (QC) measures has been applied before and after imputation. These measures have been developed by the eMERGE Genomics Workgroup [21, 22]. The standard QC process included sample call rates, sample relatedness, and population stratification, sex inconsistency as well as marker quality (i.e., marker call rate, minor allele frequency (MAF), and Hardy-Weinberg equilibrium (HWE). In this study, all analyses were limited to participants with call rates > 98%, SNPs with call rates > 99%, and SNPs with MAF > 1% and HWE *p* > 0.0001 in controls. The details of imputation process and principal component (PC) analyses have been included in Additional file 2 [23–25].

### Statistical analyses

Logistic (case-control) and quantitative linear (case-only) regression analyses were performed using an additive genetic model adjusting for 10 medical centers; PCs 1, 2, and 3; sex; and age. In addition, since NAFLD is closely linked to obesity, we included the most recent BMI for each subject as another covariate and remove all missing participants from analyses. Traditionally absolute BMI (kg/m^2^) is used for adults, while age- and sex-specific BMI-*z* scores and percentiles apply in children and adolescents to account for their continuing growth. In combined analyses, we therefore transformed all BMI into 6 classes: underweight (< 18.5 or < 5th percentile), normal (18.50–24.99 or 5th to < 85th percentile), overweight (25.00–29.99 or 85th to < 95th percentile), and obese: class 1 (30–34.99 or 95th to < 120% of the 95th percentile), class 2 (35–39.99 or 120% to < 140% of the 95th percentile), and class 3 (≥ 40 or ≥ 140% of the 95th percentile). The percentage of BMI ≥ 95% in pediatrics participants was estimated using the CDC-based online resource [27]. The distribution of participants that we received from the network also varied per site (see Additional file 1: Table S1); we therefore adjust for 10 study sites. Adjusting for too many covariates may sometimes cause the standard logistic regression to fail to converge especially for less-frequent variants. Firth’s penalized likelihood approach, available in second generation of PLINK, is a method of addressing issues of separation and bias of the parameter estimates in which we used in regression analyses when necessary [26]. For liver enzymes (AST, ALT), we used the highest value U/L per subject. All quantitative phenotypes including liver enzymes and NAS score were standardized to mean of zero and variance 1 using PLINK.

Further conditional analyses and pairwise SNP × SNP interactions were also performed using “epistasis” option in PLINK. In this study, we only analyzed the pairwise interaction effect of one known SNP (rs738409) in *PNPLA3* gene against the genome. The slower “—epistasis” command was used to test for epistasis using logistic regression which is the most accurate test to define SNP × SNP interactions [26]. Interactions were excluded if two SNPs were located within 1 Mb of each other to avoid spurious evidence of interaction due to linkage disequilibrium (LD). Narrow-sense heritability was also estimated using an SNP-based approach available in the GCTA program [28] which evaluates the proportion of phenotypic variance explained by all SNPs. Briefly, the GCTA analysis consists of two steps. First, all SNPs are used to calculate the genetic relationship matrix (GRM) among participants using the observed low-level genetic similarity in SNP data from individuals who are not directly related. This measure is then used as a predictor in a mixed linear model with a trait as the response to estimate *h*^2^ [28]. The weighted genetic risk score (GRS) was also calculated using PLINK-score function by multiplying each *β*-coefficient of highly significant SNPs with the number of corresponding risk alleles (0, 1, or 2) and then summing the products ( [26]. For known variants, *β*-coefficients were obtained from the GWAS catalog [29]. The performance of the obtained GRS score for disease diagnosis and prediction accuracy were evaluated using receiver operating characteristic (ROC) curve, using MedCalc software [30]. Finally, to estimate the level of heterogeneity between pediatrics and adult cohorts, Cochran’s *Q* test statistics was applied using PLINK2 [26].

### PheWAS analyses

A phenome-wide association study (PheWAS) was also performed in order to evaluate pleotropic effects of the known GWAS variant (rs738409) as well as other novel effects in this study with any other trait in children or adults. The trait definition in PheWAS approach is mainly based on billing International Classification of Diseases (ICD) codes; therefore, it is less conservative. The detail of this approach has been described previously [31]. We used the PheWAS package in R version 3.5.1 [32]. Briefly, in the PheWAS process, first the ICD-9 codes are collapsed into PheWAS codes according to PheWAS map [32]. Then, cases and controls are determined according to the code under study. In these analyses, a case was defined as having at least two occurrences of the PheWAS code on different days and the controls with no instances. Additionally, we used a threshold of at least 20 cases for the code to be used in the model. Next, for each PheWAS code, a logistic regression model was created and adjusted for age, sex, BMI, site of genotyping, and PCs similar to GWAS study. A false discovery rate (FDR) of 0.05 using the Benjamini–Hochberg procedure implemented in PheWAS was then used to correct the threshold for multiple hypotheses testing.

### Post-GWAS analyses and data visualization

The details of post-GWAS analyses including functional annotation, prioritization, and interpretation of GWAS results based on functional mapping are included in Additional file 2 [33–41].

### Power analyses

We used QUANTO for power calculation of case-only and case-control GWAS analyses [42]. For quantitative NAS-score analysis with 235 participants, given the mean and standard deviation of our continuous variable, i.e., NAS score (mean 3.78, SD 1.76) (see Table 1), we tested the power assuming an additive genetic model. For variants with minor allele frequency above 0.2 and effect size (βG) of at least 0.5, this sample size will have > 0.80 power to identify the association at an alpha level of 0.05. Of note, almost all of our top genetic associations for NAS score or fibrosis had minor allele frequency above 0.2. In case-control GWAS analyses with 1106 cases and 8571 controls, we had more than 90% power to detect effects for all variants with MAF > 0.01 under an additive model.

**Table 2:** The demographic distribution of EMR-linked eMERGE cohorts

	Case_EA	Control_EA	Mean age	♂/♀	Mean BMI, kg/m^2^
Pediatrics*	396	846	13.05 (SD 5.41)	693/549	22.70 (SD 7.87) †
Adults	710	7725	63.50 (SD 16.86)	3810/4625	32.64 (SD 8.21)
Total	1106	8571			

*Defined as ≤ 21 years old

†The average BMI-for-age *z* score in pediatric cohorts was 1.16 (95% CI = 1.03–1.20, SD = 1.39)

## Results

The results reported below consist of overall NAFLD case-control GWAS and four additional case-only GWA quantitative studies for NAS score, fibrosis, and AST and ALT liver enzymes.

### NAFLD case-control GWAS

Table 2 shows demographic characteristics of patients and controls included in this study. The mean age was 63.5 (±16.86 SD) for adult participants (*N* = 8435) and 13.05 (±5.41 SD) for pediatric participants (*N* = 1242). The number of participants per site is included in Additional file 1: Table S1. In this study, 47% of pediatric participants and 42% of adults were males. A total of 9677 unrelated European ancestry participants (1106 cases and 8571 controls) and 7,263,501 autosomal variants were evaluated for this GWAS analysis.

**Table 3:** Major SNP association results with NAFLD (case-control), and 4 quantitative case-only GWA studies (NAS score, fibrosis, liver enzymes ALT and AST) in the eMERGE Network. All results adjusted for age, gender, site of genotyping, 3 first principal components, and BMI. For more details and results with *p* < 10^− 5^, see Additional file 1: Table S2

NAFLD-GWAS									
SNP	CHR	Position^a^	Gene	Minor allele	MAF^b^	OR	L95	U95	*p*
rs738409	22	44,324,727	*PNPLA3*	G	0.23	1.79	1.58	2.02	1.70 × 10^−20^
rs738408	22	44,324,730	*PNPLA3*	T	0.23	1.79	1.58	2.02	1.93 × 10^−20^
rs3747207	22	44,324,855	*PNPLA3*	A	0.23	1.78	1.58	2.02	2.63 × 10^−20^
rs2294915	22	44,340,904	*PNPLA3*	T	0.25	1.75	1.55	1.97	1.40 × 10^−19^
rs2980888	8	126,507,308	*TRIB1*	T	0.31	1.36	1.20	1.53	5.98 × 10^−07^
rs2954038	8	126,507,389	*TRIB1*	C	0.31	1.35	1.20	1.52	8.30 × 10^−07^
NAS score									
SNP	CHR	Position	Gene	Minor allele	MAF	Beta	SE	*p*	
rs5748926	22	17,649,774	*IL17RA*	T	0.34	0.91	0.16	3.81 × 10^−08^	
rs738409	22	44,324,727	*PNPLA3*	G	0.41	0.85	0.15	3.94 × 10^−08^	
Fibrosis									
SNP	CHR	Position	Gene	Minor allele	MAF	Beta	SE	*p*	
rs698718	16	68,560,185	*ZFP90-CDH1*	A	0.23	0.83	0.12	2.74 × 10^−11^	
rs1645976	16	68,563,509	*ZFP90-CDH1*	T	0.23	0.83	0.12	2.79 × 10^−11^	
rs72943235	2	88,500,646	*FABP1*	A	0.01	2.38	0.43	8.18 × 10^−08^	
ALT liver enzyme									
SNP	CHR	Position	Gene	Minor allele	MAF	Beta	SE	*p*	
rs206833	2	31,708,616	*XDH*	A	0.17	0.26	0.05	3.41 × 10^−07^	
rs2294915	22	44,340,904	*PNPLA3*	T	0.34	0.20	0.04	4.04 × 10^−07^	
rs738409	22	44,324,727	*PNPLA3*	G	0.33	0.20	0.04	4.68 × 10^−07^	
AST liver enzyme									
SNP	CHR	Position	Gene	Minor allele	MAF	Beta	SE	*p*	
rs10272006	7	21,520,132	*SP4*	G	0.33	0.25	0.04	5.83 × 10^−09^	
rs7796796	7	21,499,857	*SP4*	A	0.32	0.25	0.04	6.29 × 10^− 09^	
rs62141163	2	31,663,114	*XDH*	A	0.11	0.34	0.07	2.30 × 10^−07^	

*Abbreviations*: *MAF* minor allele frequency, *OR* odds ratio, and 95% confidence interval (CI), *Beta* change in quantitative case-only phenotypes (NAS score, fibrosis (235 cases), ALT and AST liver enzymes (1075 cases)) per copy of minor allele (direction of beta is for minor alleles, *SE* standard error of beta; ^a^Position = GRch37/hg19; ^b^The direction of all effects is for the minor allele. The minor allele frequency for case-only GWA results is for cases

### Associations of previously reported SNPs

**Fig. 1:**
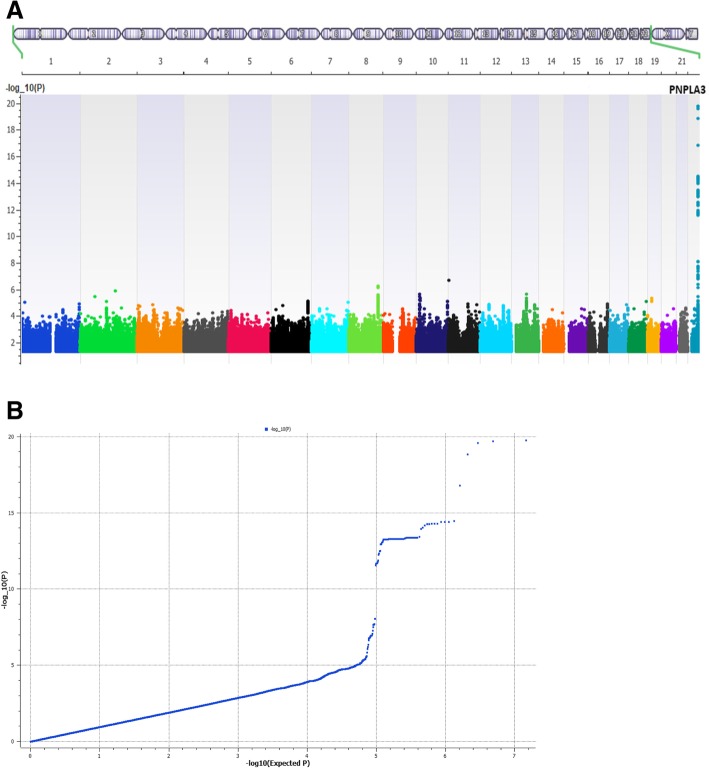
**a**, **b** Manhattan plot (**a**) and Q–Q plot (**b**) of genome-wide markers for NAFLD in European ancestry (1106 cases and 8571 controls). A total of 1106 cases of NAFLD and 8571 controls were analyzed after quality control. Logistic regression analysis was performed for 7,261,527 variants with MAF > 1% assuming an additive genetic model, adjusted for age, sex, BMI, genotyping site, and genetic ancestry (principal components 1 through 3). Results are plotted as –log10 *p* values on the *y*-axis by position in chromosome (*x*-axis) (NCBI build 37)

**Fig. 2:**
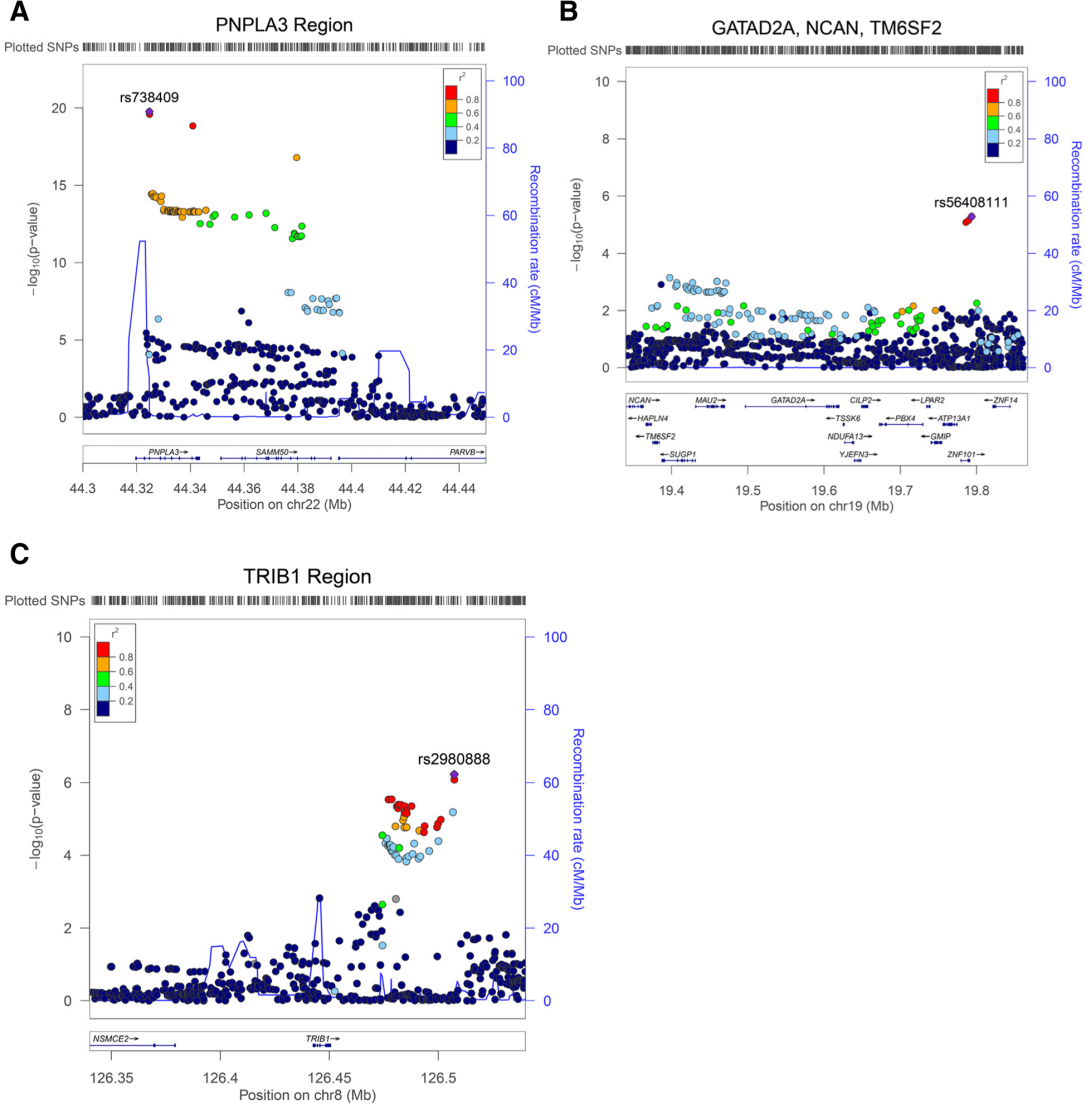
**a–c** LocusZoom plot of the associations signals in three previously known regions for NAFLD. **a** Confirmation at 22q13 for *PNPLA3*. SNP rs738409 is a missense variation (I148M) in *PNPLA3* produced the best effect (*p* = 1.70 × 10^− 20^). **b** Detected signal at 19p12 (*GATAD2A*, *NCAN*, *TM6SF2*) region. The best marker in this study was rs56408111 (*p* = 5.26 × 10^− 6^). The linkage disequilibrium (LD) between rs56408111 and previously known SNP rs4808199 was *r*^2^ = 0.24, *D*’ = 0.74. **c** Detected signal at 8q24 (*TRIB1*) genetic region. The best marker in this study (rs2980888) is shown (see also Additional file 1: Table S2). Estimated recombination rates (from HapMap) are plotted in cyan to reflect the local LD structure. The SNPs surrounding the most significant variant are color-coded to reflect their LD with the index SNP (taken from pairwise r2 values from the HapMap CEU database, www.hapmap.org). Regional plots were generated using LocusZoom (http://csg.sph.umich.edu/locuszoom)

Consistent with previous reports, we identified strong genetic signals at the *PNPLA3* locus at 22q13. Figure 1a shows a Manhattan plot with one main peak located on chromosome 22 that was associated with NAFLD. The Q–Q plot of this GWAS is also shown in Fig. 1b. The overall low inflation rate of *λ* = 1.001 indicated no major population stratification. At the *PNPLA3-SAMM50* region, three proxy SNPs (*r*^2^ > 0.95), rs738409, rs738408, and rs3747207, located in the *PNPLA3* gene showed the strongest associations (best SNP rs738409 *p* = 1.70 × 10^− 20^, OR = 1.79 (95% CI = 1.58–2.02)) (Table 3, Fig. 2a). This effect was consistent in both pediatric (*p* = 9.92 × 10^− 6^, OR = 1.76 (95% CI = 1.37–2.27)) and adult (9.73 × 10^− 15^, OR = 1.79 (95% CI = 1.55–2.08)) cohorts and with no evidence of heterogeneity (Cochran’s *Q* = 0.78, *I*^2^ = 0). Consistent with previous results, another coding variant rs2294918 (E434K) in *PNPLA3* gene was associated at a weaker level (*p* = 1.90 × 10^− 5^). The SNPs with the most significant evidence for association are summarized in Table 3, and all results with *p* < 10^− 5^ are included in Additional file 1: Table S2.

**Table 1:** Laboratory, clinical, and histologic characteristics of NAFLD patients included in the case-only association analyses. All individuals were of European ancestry

	Pediatrics	Adult	Overall
Histologic characteristic—NAS score (0–8) †	4.01(SD 1.58)	3.45(SD 1.74)	3.78(SD 1.76)
NAS score ≥ 5	43/107 (40%)	36/128 (28%)	79/235 (34%)
Histologic characteristic—fibrosis score (0–4) †	0.71(SD 0.67)	1.01(SD 1.26)	0.88(SD 1.06)
ALT U/L‡	40 (37–45)	63 (59–67)	53 (49–58)
AST U/L‡	45 (42–48)	39 (37–41)	41 (39–43)
Presence of cirrhosis	*N* = 0	*N* = 64	*N* = 64
Presence of hepatocellular CA	*N* = 0	*N* = 15	*N* = 15

†NAS and fibrosis score were available for 235 subjects (107 pediatrics and 128 adult subjects). For histologic score, mean and standard deviation is shown

‡ ALT and AST lab values were available for 1075 of cases. Medians and 95% CI of medians are shown

Apart from the main effect at the *PNPLA3* locus, previous genetic studies identified several minor effects at other chromosomal loci, including *GCKR* at 2p23, and *GATAD2A*, *NCAN*, and *TM6SF2* at 19p12 [43, 44]. We examined whether or not the associations were reproduced in our cohorts by extracting genotype information of SNP markers corresponding to these loci. None of these effects reached genome-wide significance. In our pediatric cohorts, the association of rs1260326 and rs780094 in *GCKR* was borderline significant (*p* = 0.006, OR = 1.40, 95% CI 1.1–1.78). However, the association was lost when examined in the adult cohort (see Additional file 1: Table S3). At the 19p12 region (*GATAD2A*, *NCAN*, *TM6SF2*), the association with known SNP rs4808199 was also detected using all cohorts (*p* = 0.004, OR = 1.22, 95% CI 1.06–1.40)). Of note, the known *TM6SF2* missense variant rs58542926 (E167K) produced a *p* = 0.03 (OR = 1.23, 95% CI 1.01–1.52) in our cohort. In this region, we also found other unreported downstream markers with stronger associations (best effect for SNP rs56408111 *p* = 5.26 × 10^− 6^) (see Additional file 1: Table S2; Fig. 2b). The observed effect for rs4808199 or rs58542926 disappeared after conditioning on rs56408111 (*p* = 0.71, *p* = 0.17 respectively) suggesting that the association in this region mostly derives from rs56408111. The LD between these two known markers (rs4808199, rs58542926) and the best variant in this study, rs56408111, was modest (*r*^2^ = 0.25, *r*^2^ = 0.40 respectively).

We also confirmed an effect at 8q24 near the *TRIB1* gene that previously associated with NAFLD in the Japanese population [45]. In their population, rs2954021 produced *p* = 4.5 × 10^− 5^. In our European ancestry population and for the first time, this variant as well as a cluster of variants nearby was associated with NAFLD with the best marker rs2980888 (*p* = 5.98 × 10^− 7^, OR = 1.36 95% CI = 1.20–1.53) (see Table 3, Fig. 2c). Conditional analyses suggest that rs2980888 is the most informative variant in this region in European ancestry. These two markers resided in one risk haplotype in European ancestry with (*r*^2^ = 0.45, *D*’ = 0.97) (Fig. 2c).

### Controlling for the main effects at *PNPLA3*

We used logistic regression models conditioned on the main effect at *PNPLA3* as well as testing for epistatic interaction between the known SNP at *PNPLA3* rs738409 and the rest of genome. For conditional analysis, the genotype data of rs738409 in dosage format (0, 1, 2) was included as another covariate in addition to age, sex, PCs, BMI, and sites of genotyping. While this variant controlled all effects at *PNPLA3* indicating no other independent effects at this locus, no major changes have been detected in other loci. Next, in a separate analysis, the epistatic effect of the known SNP rs738409 with the rest of the genome was evaluated (see “Methods”). Several suggestive results were detected across the genome with only one effect at 16p12 that passed the significance threshold of *p* < 0.0001 (SNP rs2188761, case-only *P*_epistasis_ = 2.47 × 10^− 7^, case-control *P*_epistasis_ = 7.32 × 10^− 6^, OR of interaction = 1.50). Several proxy markers in this region (16p12) such as rs7499477, rs2188760, and rs6497497 (r^2^ > 0.95 with rs2188761) also interact with rs738409 in *PNPLA3*. In addition, this novel epistatic effect was consistent in both pediatrics and adult cohorts (OR of interaction = 1.57 and 1.43 for pediatrics and adults, respectively). As mentioned above, all these markers had passed QC and were in HWE. However, none of these markers at the 16p12 region were genome-wide significant in GWAS analyses (0.1 > *p* > 0.02).

Because NAFLD is closely linked to obesity, we also explored the specific SNP × SNP interaction of the major obesity locus, *FTO* (rs1421085) and *PNPLA3* (rs738409). We did not find any significant SNP × SNP interaction (*p* = 0.72). Of note, the GWAS effect for *FTO* (rs1421085) in this study was *p* = 0.25 after controlling for BMI. However, by relaxing the model and removing the BMI as a covariate, this effect in *FTO* increased in significance (*p* = 9.26 × 10^− 6^).

### Case-only GWA studies

#### Impact of SNPs on the severity of NAFLD

**Fig. 3:**
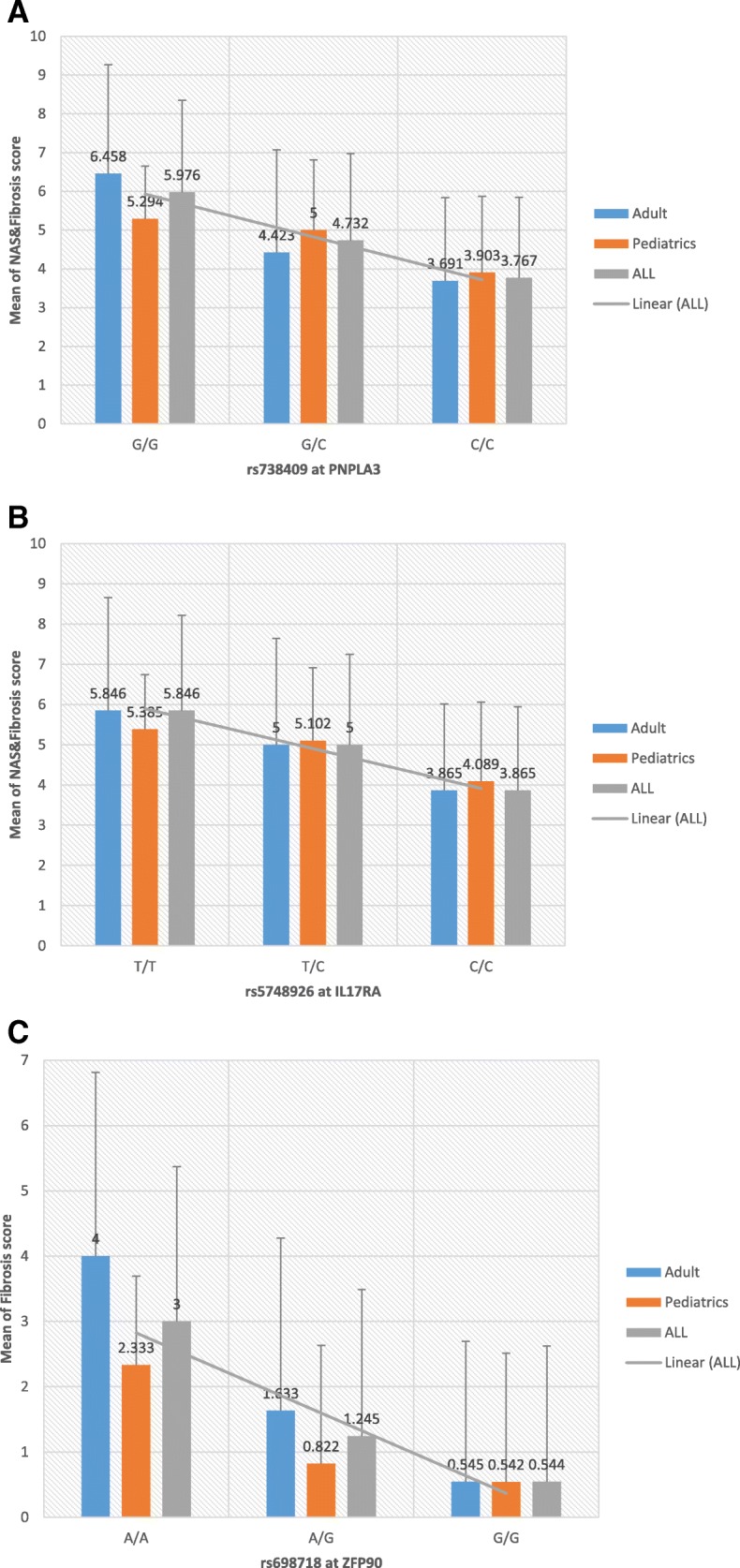
**a** Means and standard deviations of NAS and fibrosis score (0–12) stratified by genotype of rs738409 at PNPLA3 in 235 NAFLD cases. The results are plotted as the sum of NAS and fibrosis score (0–12) (*y*-axis) against the three genotypes of rs738409 C>G polymorphism (*x*-axis). The results are further sub-divided by age groups (pediatrics, adult, and all). Results for IL17RA (**b**) and ZFP90 (**c**) also are shown

We next investigated the associations of the SNPs with NAFLD disease severity based on available histopathologic grade, namely, NAS and liver enzymes (see “Methods”). Because liver biopsy usually is not indicated for NAFLD diagnosis, we were able to identify and score only 235 participants using EMR data from the total of 1106 NAFLD cases that includes 107 pediatric and 128 adult cases (Table 1). Liver function tests (AST U/L, ALT U/L) were available for 1075 of case participants. In addition to the main case-control study, for disease severity index, we performed GWAS for each quantitative trait (NAS score, fibrosis, ALT, AST) using linear regression method adjusting for age, sex, BMI, PCs, and site of genotyping. These case-only analyses showed several loci with significant associations. The SNPs with the most significant evidence are summarized in Table 3 and all results with *p* < 10^− 5^ are provided in Additional file 1: Table S2. Consistent with previous reports, index SNP rs738409 at *PNPLA3* showed a significant association with disease severity NAS score (*p* = 3.94 × 10^− 8^, beta = 0.85) (Table 3). Indeed, if we consider a binary outcome in which NAS score ≥ 5 as case versus the remaining cases as control (79 cases versus 156 controls), an OR = 2.72, 95% OR (1.83–4.04), and *p* = 4.27 × 10^− 7^ can be obtained for this marker. As shown in Table 1, 34% of our participants had a NAS score ≥ 5. Figure 3 also shows the mean of NAS score and fibrosis together (0–12) stratified by *PNPLA3* index SNP rs738409-genotype (GG/GC/CC) in which a beta of 1.07 (SE = 0.20) can be obtained. This is almost equal to one unit increase in NAS scores per risk allele.

Apart from the main effect at the *PNPLA3* locus that also was associated with disease activity, previous genetic studies also identified several effects for histologic NAS score, fibrosis, and liver enzyme in NAFLD cases [10, 46]. In particular, Chalasani et al. evaluated 236 well-characterized NAFLD European ancestry female cases using 324,623 SNP markers for the histologic traits. After extracting genotype information of SNP markers corresponding to these loci, none of these effects reached genome-wide significance in our cohort. However, an effect for SNP rs1227756 at *COL13A1* was associated (*p* = 0.008) with the NAS score (Additional file 1: Table S3). Another published effect was at chromosome 8 for SNP rs2645424 near *FDFT1* for NAS score; however, a subsequent study failed to confirm it [47]. While association with this marker was weak in our cohort (best *p* = 0.15 for fibrosis), several nearby markers in this region were suggestively associated including eQTL variant rs1908814 (best *p* = 1.49 × 10^− 4^) for the same trait but with low LD with the previously published marker (*r*^2^ = 0.01) (see Additional file 1: Table S3). A recent study also identified an association of a splice variant in *HSD17B13* (rs72613567:TA insertion) with reduced risk of NAFLD (*p* = 1.3 × 10^− 5^) [48]. In our main case-control GWAS analysis, while we detected a trend of association with this indel in the same direction, it was not significant after adjusting for covariates (Additional file 1: Table S3). Of note, another reported missense variant in this gene (rs62305723) which encodes a P260S substitution, was weakly associated in the pediatric only cohort (*p* = 0.05) (Additional file 1: Table S3) [49]. Additionally, in NAS score analysis in this region, we detected a novel eQTL marker for *HSD17B13* (rs3923441) that was nominally significant with NAS score (*p* = 0.008, beta = 0.55) (Additional file 1: Table S3) and produced a PheWAS effect for abnormal liver enzyme levels (see the “PheWAS approach” section). Of note, the LD between these markers was weak (r^2^ < 0.1). We also evaluate whether any of the implicated HSD17B13 allele modifies the risk of liver injury associated with PNPLA3 rs738409 by SNP × SNP interaction analyses. While all results were suggestive, we observed a nominally significant interaction effects between rs3923441 and rs738409 with AST level (*p* = 0.01, beta interaction = 0.19) as well as ALT level (*p* = 0.03, beta interaction = 0.16). Of note, these two effects were improved if we included only obese persons (for AST *p* = 0.002, beta interaction = 0.24, and for ALT *p* = 0.02, beta interaction = 0.18 respectively).

#### Novel effects

**Fig. 4:**
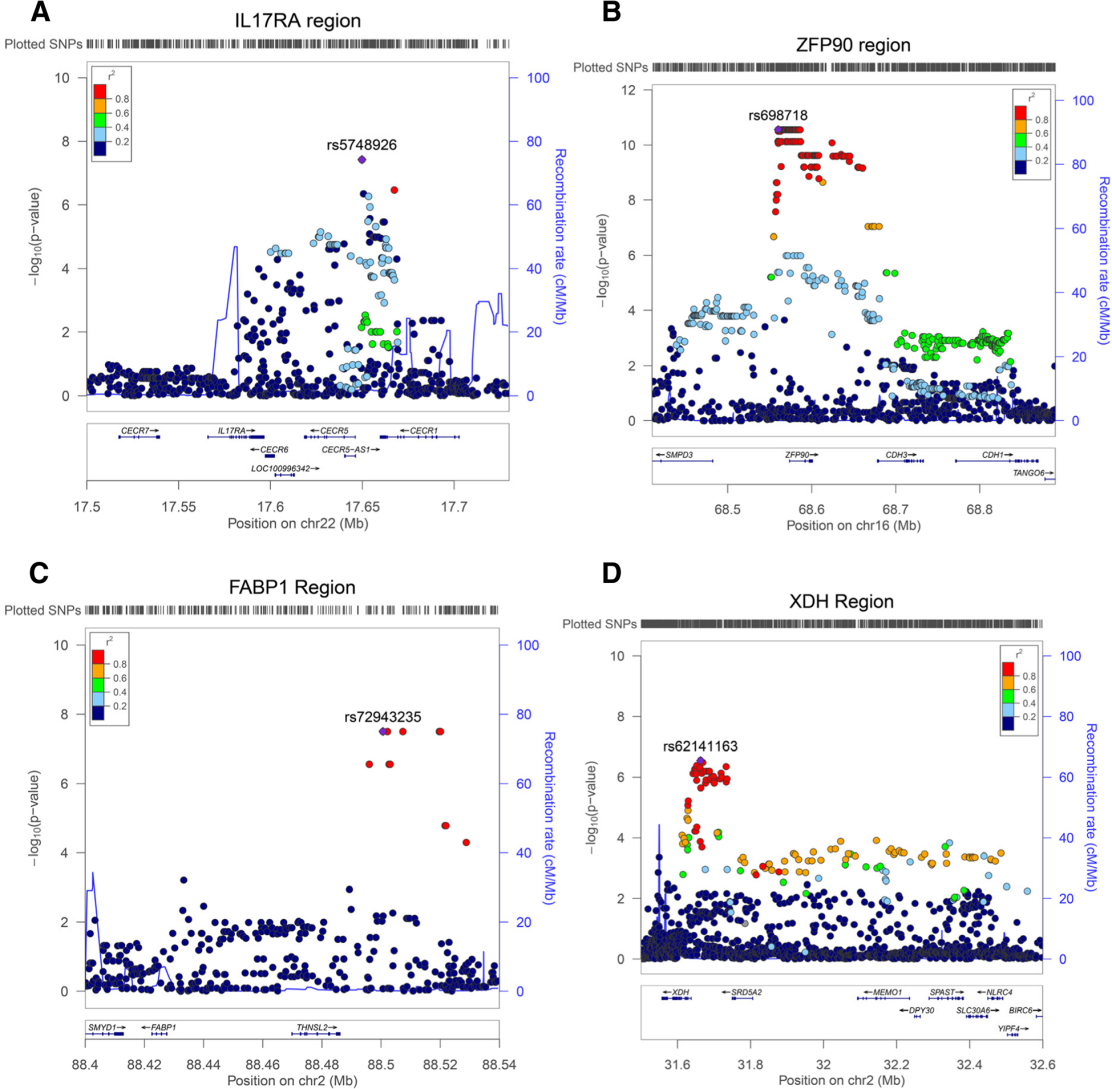
**a–d** Regional association plots of best effects in case-only linear regression analyses for continuous traits of NAS score, fibrosis, and ALT liver enzyme, respectively. **a** The best observed effect near the IL17RA region for NAS score. **b** The most significant effects at 16q22 near *ZFP90* gene for fibrosis. **c** The effect near *FABP1* locus for fibrosis. **d** An effect at 2p22 near *XDH* for AST liver enzyme

Across the genome, we identified several new effects that have not been reported previously and evaluated the nearby functional markers at *r*^2^ > 0.6 (Fig. 4a–c). Indeed, a few of them reached genome-wide significance levels (*p* < 5.0 × 10^− 8^), including a novel effect for NAS score at 22p13 in which a cluster of SNPs near *IL17RA* were associated; best SNP = rs5748926, *p* = 3.81 × 10^− 8^, beta = 0.91 (Fig. 4a, Table 3). For fibrosis, a novel effect was detected at 16q22 near the *ZFP90* locus (best SNP rs698718, *p* = 2.74 × 10^− 11^, beta = 0.83) (Fig. 4b, Table 3). There was no evidence of heterogeneity between pediatrics and adult for these two new effects (Cochran’s *Q* = 0.24 and 0.37) respectively. Of note, the SNP × SNP interaction effects between rs738409 (*PNPLA3*) and either rs5748926 (*IL17RA* region) or rs698718, (*ZFP90* region) were suggestive or not significant (*p* = 0.02 and *p* = 0.61 respectively). Another significant effect was detected on the short arm of chromosome 2 near the *FABP1* gene. The best marker, rs72943235, produced a *p* = 8.18 × 10^− 8^ for fibrosis and *p* = 3.17 × 10^− 8^ for NAS score plus fibrosis (Fig. 4c, Table 3); however, most of the variants in this cluster were rare in the European ancestry participants (1% < MAF < 5%, see Additional file 1: Table S2). More common markers in this region such as rs4618056 had a weaker GWAS effect (*p* = 0.0004) and did not show significant LD with rs72943235 (*r*^2^ = 0.05).

We also evaluated transaminase test (AST and ALT) levels as a surrogate quantitative biomarker for NAFLD disease activity. The median values of AST and ALT were 41 and 53 respectively among NAFLD cases (see Table 1). As expected, higher values of the NAS score were associated with higher levels of ALT and AST in 216 overlap participants (*p* = 0.001, correlation coefficient *r* = 0.23). Analyses of AST or ALT levels in 1075 cases showed a robust association at *PNPLA3* (best effect was for ALT rs738409 *p* = 4.68 × 10^− 7^, beta = 0.20, Table 3) again indicating the importance of *PNPLA3* for disease severity and higher liver enzyme levels. In addition, a common novel effect at 2p22 near the *XDH* gene can be detected for both AST and ALT. The best variant rs62141163 produced a *p* = 2.30 × 10^− 7^, beta = 0.34 for AST (Fig. 4d, Table 3). Some of the effects were more specific to individual liver enzyme (AST or ALT). An effect at 7p15 in the *SP4* transcription factor (best marker rs10272006 *p* = 5.83 × 10^− 9^, beta = 0.25, Table 3) was observed for the AST enzyme level, and an effect near *SDC1* (rs6531222, *p* = 5.16 × 10^− 6^, beta = 0.18, Additional file 1: Table S2) was identified for the ALT liver enzyme. We summarized all suggestive genetic effects regarding disease severity, i.e., NAS score, fibrosis, and liver enzymes (*p* < 10^− 5^) in Additional file 1: Table S2.

End-stage liver disease is another measure of disease severity and outcome. In this cohort, there were 64 adult participants with liver cirrhosis (15 of them with hepatocellular cancer, see Table 1). As expected, a higher effect size for rs738409 at *PNPLA3* was obtained when only NAFLD plus presence of cirrhosis were compared with healthy controls (OR = 2.0, 95% CI 1.38–2.86, *p* = 0.0001).

#### Gene-based and pathway analyses

We annotated the most significant variants in this study (including SNPs in LD), for cis-eQTL effect and other regulatory functions and report in brief in Additional file 1: Table S4. We also provide the average direction of gene expression based on the risk alleles in several related tissues including blood, skin fibroblast, adipocytes, liver, and gastrointestinal tissues according to GTEx (v7). In Additional file 1: Table S4, other regulatory functions from Roadmap Epigenomics including enhancer, motif change, DNAse hypersensitivity, protein bounding effects, and chromatin marks specific for the liver have been shown. For pathway enrichment analyses, first, we performed gene-based analyses using MAGMA that results in 4 genes with significant (gene-based threshold of 2.72 × 10^− 6^) and 39 genes with suggestive results (*p* ≤ 10^− 3^). Additional file 1: Table S5 shows all MAGMA gene-based result for NAFLD case-control GWAS at *p* < 0.05. Since some lead SNPs are quite remote from the associated gene transcripts, we also separately annotated and identified all functional SNPs with GWAS *p* ≤ 10^− 5^ and assigned a gene to a locus if the index SNP or linked variants (*r*^2^ > 0.6) have any functional effect on that gene (see “Methods”). We combined this gene list with MAGMA gene-based results mentioned above for a total of 79 genes to be evaluated for pathway enrichment. Gene sets available in the Molecular Signatures Database (MSigDB) that are divided into 8 major collections (C1-C8) were primarily used for pathway-based analyses. After Bonferroni correction, several pathways were enriched including Intrleukin-1 receptor binding genes (*p* = 8.05 × 10^− 17^) in GO molecular functions C5 (MsigDB c5) and genes in mitochondrial assembly (GO cellular components) (*p* = 4.51 × 10^− 5^). Since several genes in the IL-1 receptor pathway were co-located at the same genomic region in chromosome 2, more restricted LD pruning (*r*^2^ > 0.2) was also applied to avoid potential inflation in enrichment analyses and results for this pathway still remained significant (*p* = 7.76 × 10^− 15^). At the 22q region, *PNPLA3* incorporate mostly in the phospholipid metabolism and lipase activity pathways, *SAMM50* enriched in the mitochondrial assembly pathway (GO cellular components), and *PARVB* enriched in the liver cancer pathway (see Additional file 1: Table S6).

**Fig. 5:**
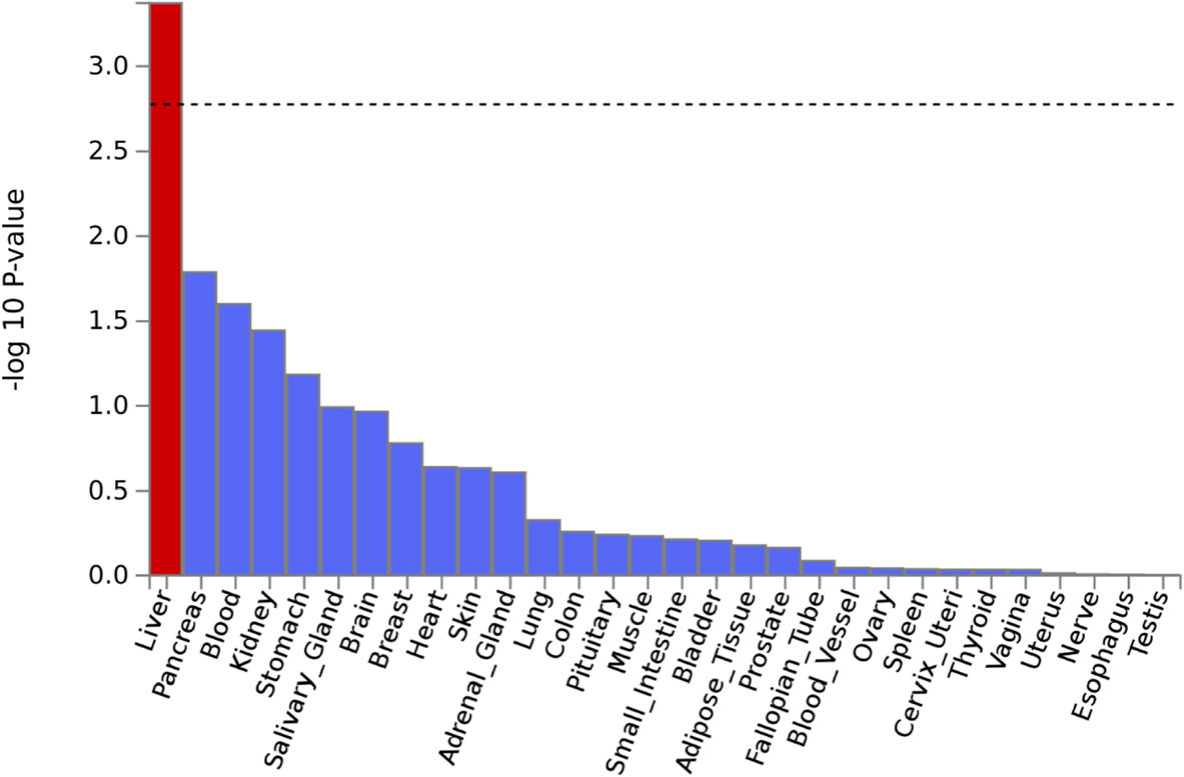
NAFLD case-control gene-based results using MAGMA as a base and tissue-specific gene expression (GTEx v7 with 30 general tissue types) as a source produced specific enrichment in liver (see “Methods”). List of all MAGMA gene-based results (*P* < 0.05) is shown in Additional file 1: Table S5

We followed the above approach for GWAS of NAS score, fibrosis, and liver enzymes, identified nominated genes for each group, and provide significant pathway enrichment results in each group and all combined for a total of 349 genes. All nominated genes by GWAS are listed in Additional file 1: Table S6. Of note, gene sets for the TGFB signaling pathway particularly showed enrichment for fibrosis and NAS score (*p* = 1.62 × 10^− 4^) and *IL17RA* was enriched in GO_receptor binding (MsigDB c5) (*p* = 1.49 × 10^− 4^) and immunologic signature (MsigDB c7) (*p* = 1.71 × 10^− 3^) (Additional file 1: Table S6). Furthermore, in order to test the relationships between tissue-specific gene expression profiles and NAFLD-gene association results, MAGMA gene-property analysis was performed using GTEx (v7) as a reference. As shown in Fig. 5, this approach particularly revealed a specific gene expression enrichment of liver tissue when applying NAFLD case-control GWAS. Similarly, according to HaploReg (V4.1), GWAS loci at *p* < 10^− 5^ as reported in Additional file 1: Table S2 were enriched with enhancer regulatory elements in liver and adipose tissue (*p* = 1.30 × 10^− 5^ for liver and *p* = 1.50 × 10^− 5^ in adipocyte). For other GWAS related to sub-phenotypes, this liver enrichment was not detected.

Next, TF-enrichment analysis was applied using Regulatory Element Locus Intersection (RELI) (see “Methods”), a novel algorithmic approach to nominating candidate regulatory variants based on LD pattern and CHIP-Seq data. In contrast to other enrichment analyses that start with a list of nominated genes, this algorithm takes a list of associated risk SNPs as an input. We applied this method for all SNPs with *p* < 10^− 5^. The list of TF for NAFLD case-control GWAS that survived the multiple test corrections is included in Additional file 1: Table S6. The top-ranked TFs include *NFIL3* (*p* = 2.95 × 10^− 16^), *PPARG* (*p* = 3.36 × 10^− 11^), *SPI1* (*p* = 1.30 × 10^− 07^), and *FLI1* (*p* = 6.52 × 10^− 04^). Moreover, in these analyses, when we limit the ChIP-seq datasets to only liver cells, a liver specific TF, *CEPBA*, was at the border of significance (see Additional file 1: Table S6). *PPARG* TF enrichment can also be detected for sub-phenotypes including NAS score with less magnitude (data not shown).

As part of FUMA module, GWAS catalog (release e89) was also used as a source to determine the genetic sharing and enrichment of all nominated genes in this study with other traits. As expected, metabolic traits, NAFLD, liver enzyme, and obesity-related traits were in the top list and provided in Additional file 1: Table S6.

#### Heritability estimate

As mentioned above, heritability estimates of NAFLD range from 20 to 70% in different family studies [7, 8]. Using SNP-based approach, and with the estimated prevalence of 0.3 of this trait in the general population, we obtained a narrow-sense heritability of *h*^2^ = 0.24, (SE = 0.03) in our cohort adjusting for all covariates including BMI. This approach, however, needs a large number of participants for accurate estimation, and therefore, standard errors were higher for smaller group-studies such as pediatrics-only participants, though with higher heritability estimate in our cohort (*h*^2^ = 0.53 (SE = 0.27)).

#### PheWAS approach

We also applied PheWAS to evaluate the pleotropic effect of the known *PNPLA3* variant rs738409 as well as novel variants in this study against available traits in all eMERGE Network participants. PheWAS is a less conservative approach in terms of phenotype definition and mainly based on ICD-9 and ICD-10 disease classification codes but provides more statistical power. The detail of methodology described in “Methods” and previous publications. All results were adjusted for the abovementioned covariates, and multiple hypotheses testing using a false discovery rate (FDR < 0.05) was implemented. In this approach, 17 traits satisfied the FDR criteria (Additional file 1: Table S6). Almost all of the significant traits were related to the spectrum of liver diseases including NAFLD, liver cirrhosis, alcoholic fatty liver condition, esophageal bleeding, and hepatocellular liver cancer. Unexpectedly, we found a negative correlation between *PNPLA3* variant rs738409 with gout or gouty arthropathy (*p* = 1.09 × 10^− 4^, beta = − 0.12, SE = 0.03) (Additional file 1: Table S6). Interestingly, this inverse association with gout remained significant after conditioning for NAFLD disease status as another covariate indicating an independent effect (*p* = 4.67 × 10^− 5^, beta = − 0.14, SE = 0.03). Of note, ICD9 codes related to viral or chronic hepatitis or psychological alcohol dependence did not show association with *PNPLA3* (hepatitis C *p* = 0.07, alcohol dependence *p* = 0.39).

In addition, PheWAS evaluation of novel variants in this study results in two significant findings: one for marker rs2980888 at *TRIB1* gene that was associated with disorders of lipoid metabolism (*p* = 8.63 × 10^− 7^) and another for novel eQTL variant rs3923441 near *HSD17B13* that was associated with an abnormal liver function test (*p* = 3.74 × 10^− 6^, see Additional file 1: Table S6). Moreover, these two effects remain significant after conditioning on NAFLD status with *p* = 2.60 × 10^− 6^ and *p* = 3.19 × 10^− 6^ respectively.

#### Genetic risk score (GRS) for disease prediction

**Fig. 6:**
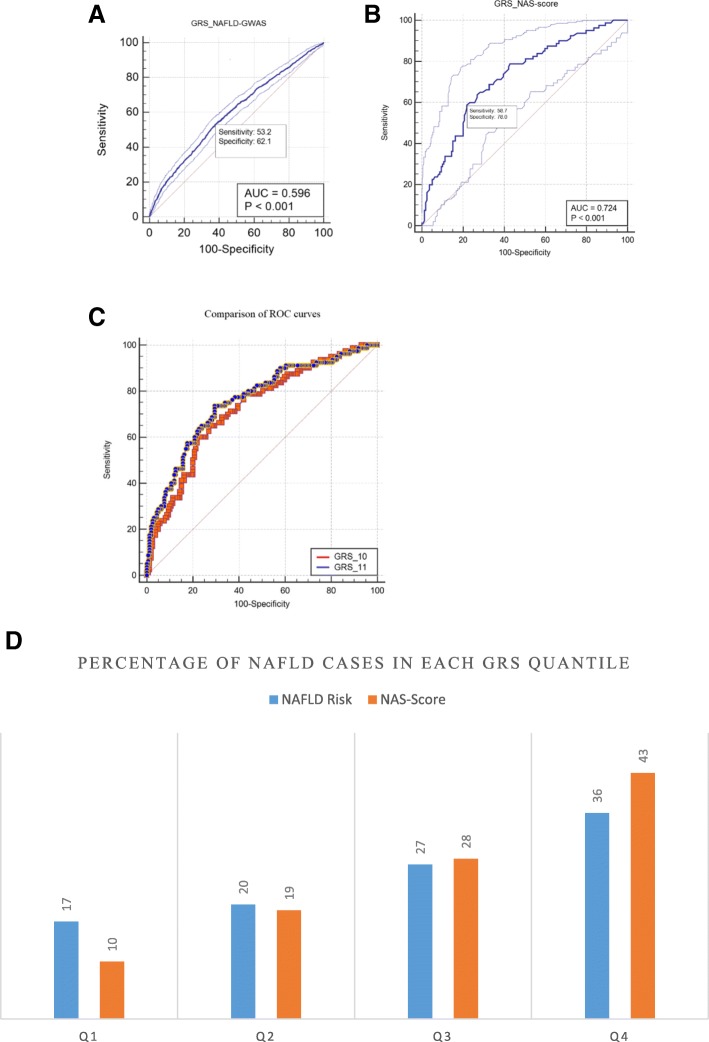
**a–d** ROC graphical plot that illustrates the diagnostic ability of the binary classifier NAFLD (cases and controls) and NAS score (above and below 5) using weighted GRS score of ten previously published SNPs (GRS-10, see “Results”). The sensitivity and specificity and AUC measures for each plot are also shown. **a** ROC curve for NAFLD-1106 cases and 8571 controls. **b** ROC curve for NAS score (79 cases above NAS score ≥ 5 versus 156 controls with score < 5). **c** Adding SNP rs5748926 near IL17RA improved the ROC curves for NAS score (GRS_11); difference between areas 0.035 (SE = 0.012, *p* = 0.004). **d** Distribution of quantiles of weighted 10-SNP GRS in NAFLD (cases and controls) and NAS score (above and below 5); percentage of NAFLD risk increases by increasing GRS quantiles; for NAFLD (cases and controls) from 17% in Q1 to 36% in Q4 (OR = 2.16, 95% CI = 1.81–2.58, *p* < 0.0001); for NAS score above 5 (defined as case) from 10% in Q1 to 43% in Q4 (OR = 8.50, 95% CI 3.45–20.96). The weighted 10-SNP GRS was calculated by multiplying the sum of the number of risk alleles (0, 1, 2) with the allele-specific effect sizes (beta coefficients) obtained from previous publications (see “Methods”)

We also calculated weighted GRS based on the known risk SNPs for NAFLD to evaluate the efficiency of this approach in eMERGE cohorts. For this purpose, we selected SNPs from previous publication in which we could also confirm at the level of *p* < 0.05 in this study and that were not in complete proxy with each other (*r*^2^ < 0.99). As a result of this criteria, ten variants (GRS-10) from genes *PNPLA3* (rs738409, rs3747207, rs2294915, rs2294918), *GCKR* (rs1260326, rs780094), *TM6SF2* (rs4808199, rs58542926), *COL13A1* (rs1227756), and *TRIB1* (rs2954021) were used to profile our case and control participants. Using this initial information, we generated ROC curves which provide a measure for the diagnostic power for both disease and disease severity. Figure 6a shows the ROC plot for prediction of overall NAFLD diagnosis (1106 cases and 8571 controls) using 10-SNPs (GRS-10) in which the area under the ROC curve (AUC) of 60% was obtained. In addition, when weighted 10-SNP GRS values were distributed according to quantiles (Fig. 6d), the prevalence of NAFLD significantly increased by increasing quantiles with a 2.2-fold increase in NAFLD risk when the highest to the lowest GRS quantiles were compared (OR = 2.16, 95% CI = 1.81–2.58, *p* < 0.0001) (Fig. 6d).

The same set of SNPs however had better performance for predicting disease severity, defined here as NAS score above and below 5 (79 cases above NAS score ≥ 5 versus 156 controls with score < 5) (AUC = 72%) (Fig. 6b). This was equal to > 8-fold increase in disease severity when the highest to the lowest GRS quantiles were compared (OR = 8.50, 95% CI 3.45–20.96) (Fig. 6d). As expected, adding novel findings from this study can improve the area under the curve respectively, and therefore, this needs to be verified in an independent population; in particular, adding only one SNP rs5748926 near *IL17RA* for NAS score (GRS-11) improved the AUC to 76% and the difference was statistically significant (difference between areas = 0.035 (SE 0.012), *p* = 0.004)) (Fig. 6c).

## Discussion

NAFLD has become the most common chronic liver disease worldwide, but currently, only limited therapies exist. A better understanding of the genetic biomarkers for this epidemic may help inform the development of novel therapeutics. The objective of this project was to develop an NLP algorithm for the NAFLD/NASH phenotype, identify cases and controls with high predictive values, and perform GWAS using data from the eMERGE Network. We demonstrate that DNA biobanks linked to EMRs can be used to identify true cases and controls for NAFLD as well as disease severity index. By using this approach, we confirmed the association of *PNPLA3* and two nearby genes (*SAMM50* and *PARVB*) for NAFLD. We also detected an additive relationship between index SNP rs738409 and disease severity in which presence of the risk allele can increase the NAS severity score approximately one unit per risk allele. This result was noted in both adult and pediatric participants and with no heterogeneity (Fig. 3). Furthermore, the epistatic effect of the known SNP rs738409 with the rest of the genome produced at least one suggestive effect near the *ACSM5* at chromosome 16p12. Acyl-CoA synthetase medium chain family member 5 (*ACSM5*) is a mitochondrial gene belonging to a family of medium chain acyl-CoA synthetases, mostly expressed in liver and fat tissues with key roles in energy storage and metabolism. Further studies are needed to validate or refute this suggestive novel epistatic effect. In this study, we did not find any significant interaction between the FTO alpha-ketoglutarate-dependent dioxygenase (*FTO*) variants (rs1421085) and *PNPLA3* (rs738409) indicating that the effect of *FTO* on pathogenesis of NAFLD is not directly dependent on *PNPLA3* genotypes but more likely by means of increasing BMI-set point.

In PheWAS analyses, we found that the SNP rs738409 is associated with a wide spectrum of liver pathologies including not only NAFLD, but also alcoholic fatty liver condition, hepatocellular liver cancer, and liver cirrhosis. In addition, this effect tends to be independent of viral hepatitis or psychological alcohol dependence. This PheWAS also indicates an inverse association between the *PNPLA3* risk allele and presence of gout. The relationship between uric acid and *PNPLA3* either in disease state or normal population has not been described previously. There has been a clear correlation between higher serum uric acid and NAFLD disease severity [50]. Although this relationship seems to be contradictory, however, uric acid is also a powerful anti-oxidant [51] and lower serum uric acid might reinforce the oxidative stress especially on early disease stages.

This study, for the first time in European ancestry participants identified an effect at 8q24 near *TRIB1* gene for NAFLD that was previously reported in the Japanese population [45]. *TRIB1* (tribbles pseudokinase 1) is highly expressed in bone marrow and liver and regulates activation of MAPK kinases and involves in regulating proliferation, apoptosis, and cytokine production. Indeed, modulation of *TRIB1* expression affects hepatic lipogenesis and glycogenesis through multiple molecular interactions [52]. Several GWAS effects have been attributed to this gene for other metabolic traits including serum adiponectin level, liver enzyme, lipid traits, and response to statin therapy [53–55]. Of note, the best marker in our European ancestry study, rs2980888, has enhancer histone mark properties in liver and several tissues (Additional file 1: Table S4). Indeed, in PheWAS analysis, we also detected an independent effect of rs2980888 for disorder of lipoid metabolism (*p* = 8.63 × 10^− 7^, Additional file 1: Table S6).

Gene-based and enrichment pathway analyses for the main NAFLD GWAS indicate an IL1 pathway as a potentially important pathway (see “Results”; adjusted *p* = 7.76 × 10^− 15^, Additional file 1: Table S6). The IL-1 family members are released upon cell death by necrosis and induce a cascade of proinflammatory cytokines resulting in sterile inflammation, a feature of NAFLD. These cytokines are also critically involved in liver inflammation, steatosis, fibrosis, and cancer development [56]. In fact, concentrations of proinflammatory IL-1 members are increased in patients with severe obesity [57].

In this study, we also identified novel variants associated with NAFLD disease severity, in particular an effect near the *IL17RA* locus for NAS score and another effect at the *ZPF90-CDH1* locus for fibrosis. *IL-17RA* is ubiquitously expressed on a wide range of tissues (liver, intestine, lung, adipose tissue) and cell types (endothelial and immune cells). Indeed, previous published findings clearly established that the IL-17 axis plays an important role in NAFLD pathogenesis in multiple NAFLD murine models [58–60] including a role in a recently described, more human-like experimental model of NAFLD [61]. Notably, our novel data reinforce these findings in humans with a promising novel genetic biomarker (rs5748926, see Additional file 1: Table S4). As shown in Additional file 1: Table S4, a decreased expression of this gene is predicted given the haplotype risk allele in our cohort. Similarly, *il-17ra*−/− mice exhibit increased obesity and hepatic steatosis when fed an obesogenic diet although they are protected from downstream inflammatory damage [59]. Because of the high correlation of the NAS score sub-components in human liver histology, additional samples are necessary to fully elucidate deeper relationships between each component of NAS histologic criteria and this variant, such as the presence of only steatosis without lobular inflammation or presence of inflammation without significant steatosis. Consistent with the murine findings, our data indicate that this effect is mainly related to steatosis-driven NAS score rather than fibrosis, and the result remained significant after conditioning on fibrosis state as additional covariate (*p* = 9.38 × 10^− 7^). The functional consequence of ZFP90 in the context of NAFLD fibrosis however is less clear. It has been previously shown that the zinc finger protein 90 (zfp90) transgenic mice had significantly increased body weight, and retroperitoneal, mesenteric, and subcutaneous fat mass [62]. In addition, genome-wide association studies have identified this region *ZFP90-CDH1* among ulcerative colitis risk loci [63]. Cadherin 1 (*CDH1*) encodes E cadherin, a transmembrane glycoprotein with a key function in intercellular adhesion in the intestinal epithelium; it also acts as a tumor suppressor protein and involved in the TGF-beta signaling pathway in which we found the nominally significant enrichment result in our fibrosis GWA study (see Additional file 1: Table S6). Another effect for fibrosis was near *FABP1* (see Fig. 4c). Most of the associated variants in this cluster however were rare in European ancestry participants (1% < MAF < 5%, see Additional file 1: Table S2 and 4). Fatty acid-binding protein (FABP) family members are involved in intracellular lipid metabolism and play roles in nuclear receptor regulation. *FABP1* is mainly expressed in the liver and at very high levels found in the cytoplasm of hepatocytes. In murine studies, *fabp* deletion attenuates both diet-induced hepatic steatosis and fibrogenesis [64]. Indeed, in human studies, serum liver fatty acid-binding protein has shown a positive correlation with NAS score (*p* = 0.03, *r* = 0.312) and fibrosis (*p* = 0.02, *r* = 0.324) [65]. A recent study also identified an association of a splice variant in one of the 17β-HSD family members, *HSD17B13* (rs72613567:TA insertion) with reduced risk of NAFLD [48]. This family of proteins plays an important role in lipid metabolism [48]. While this effect was weak in our cohort, we detected another eQTL marker for HSD17B13 (rs3923441) that was nominally significant with NAS score (*p* = 0.008, beta = 0.55), and it also showed a PheWAS effect for abnormal liver enzyme levels (*p* = 3.74 × 10^− 6^, see Additional file 1: Table S6). We also observed a nominally significant interaction effect between rs3923441 and rs738409 in PNPLA3 with AST and ALT levels especially if we included only obese persons (for AST *p* = 0.002, beta interaction = 0.24, and for ALT *p* = 0.02, beta interaction = 0.18 respectively). Interestingly, the similar findings has been reported between rs72613567:TA insertion and PNPLA3 (rs738409) for liver transaminases [48].

In case-only GWAS analyses using standardized liver enzyme as a quantitative phenotype, a robust effect at *PNPLA3* (best effect for ALT rs738409 *p* = 4.68 × 10^− 7^) was noted indicating the association of the *PNPLA3* risk allele with higher ALT levels, a biomarker for disease severity. This is also consistent with a previous publication [66]. Another common novel effect at 2p22 near the *XDH* (xanthine dehydrogenase) gene was detected for both AST and ALT. Xanthine dehydrogenase is involved in the oxidative metabolism of purines and is highly expressed in the liver. This enzyme catalyzes the oxidation of hypoxanthine to xanthine and xanthine to uric acid. Uric acid and reactive oxygen species (ROS), produced by XDH, therefore, could cause inflammation and oxidative stress. Indeed, it is recently been shown that the serum level of xanthine dehydrogenase is correlated with obesity-related metabolic indexes in blood such as triglycerides, cholesterol, and glucose [67]. An effect at 7p15 in the *SP4* transcription factor gene was also observed for AST enzyme level. SP transcription factors are overexpressed in many different cancer cell lines including hepatocellular carcinoma [68]. Among suggestive effects for liver enzyme levels, an effect near Syndecan-1 (*CD138*, *SDC1*) is noteworthy as it is a transmembrane heparan sulfate proteoglycan expressed highly in the liver and exert metabolic effects. Indeed, the serum syndecan-1 level has been shown to be increased among NAFLD patients [69]. Furthermore, transcription factor enrichment analyses using RELI nominate TF such as *PPARG* (peroxisome proliferator-activated receptor gamma) which is a master regulator of adipocyte differentiation that trans-activates multiple target genes involved in lipid metabolic pathways and inflammation. These targets include *PNPLA3* and *SAMM50* two nearby genes that we found the most significant results [70]. When we limit CHIP_seq experiments only to liver cells, another liver-specific TF (*CEPBA*) was enriched. Likewise, *CEPBA* (CCAAT/enhancer binding protein alpha (C/EBP)) is essential for the regulation of hepatogenesis, adipogenesis, and hematopoiesis. Overall, our post-GWAS association strategy combined with enrichment analyses invokes several novel associations that require further studies to elucidate the biological basis for these initial findings.

### Strengths and limitations

The major strengths of our study include stringent quality control in both genotypic and phenotypic data and minimal population stratification. In genomic analyses, we explored both case-control and case-only GWA studies for NAFLD and nominate more than 300 genes. We attempted to increase emphasis toward functional annotation and downstream genomic dissection using additional bioinformatics tools available in public resources. Another strength of our study is that the eMERGE cohorts represent many geographic areas in USA and include both adolescents and adults. Indeed, all of the main results in this study consisted in both adolescent and adult cohorts. However, other ancestry groups are under-represented in the eMERGE Network, especially after NLP processing and sub-phenotyping. Electronic medical records have a potential for unintended health errors in billing codes, lab measures, and clinical diagnoses. We have controlled and removed outliers and exclude confounding medical diagnoses using NLP processing such as alcoholic liver condition, viral hepatitis, and others to avoid potential biases. Nonetheless, the quantitative traits such as circulating levels of liver enzymes that are widely used as indicators of liver disease are not specific, and the results we provide here need to be replicated in larger cohorts in the context of NAFLD. The associations with *PNPLA3* for both NAFLD and disease severity were highly consistent with previous publications that have recruited well-characterized participants, thus serving as validation of our overall approach.

## Conclusion

In summary, we report genome-wide significant loci associated with NAFLD and disease severity index in a GWAS analysis of 9677 European ancestry individuals from 10 eMERGE study cohorts. Apart from the *PNPLA3* effect, the GWAS implicates *IL17RA* and other biologically informative genes as important contributors to disease severity of NAFLD. The results also highlight strong overlap of *PNPLA3* in the genetics of NAFLD and other liver pathologies and metabolic traits in the population, indicating a spectrum of conditions.

## Additional files

Additional file 1: One excel file with 6 master tables divided into 18 table-sheets. (XLSX 227 kb)

Additional file 2: Additional methodology. (DOC 53 kb)

## Data Availability

Genetic data for the eMERGE Network is available from the coordinating center and can be accessed through dbGAP (phs000888.v1.p1).

## Abbreviations

AASLD: American Association for the Study of Liver Diseases
*ACSM5*: Acyl-CoA Synthetase Medium chain family member 5 (gene)
ALT: Alanine aminotransferase
AST: Aspartate aminotransferase
AUC: Area under the ROC curve
BMI: Body mass index
CADD: Combined Annotation-Dependent Depletion
*CDH1*: Cadherin 1 (gene)
*CEPBA*: CCAAT/enhancer binding protein alpha (C/EBP) (gene)
*COL13A1*: Collagen type XIII alpha 1 chain (gene)
eMERGE: The electronic MEdical Records and GEnomics Network
EMR: Electronic medical record
*FABP1*: Fatty acid-binding protein 1 (gene)
FDR: False discovery rate
*FLI1*: Fli-1 proto-oncogene ETS transcription factor (gene)
*FTO*: FTO alpha-ketoglutarate dependent dioxygenase (gene)
FUMA: Functional mapping and annotation of genome-wide association studies
*GATAD2A*: GATA zinc finger domain containing 2A (gene)
*GCKR*: Glucokinase regulator (gene)
GRS: Genetic risk score
GTEX: Genotype-Tissue Expression
GWAS: Genome-wide association study
HRC: Haplotype Reference Consortium
*HSD17B13*: Hydroxysteroid 17-beta dehydrogenase 13 (gene)
HWE: Hardy-Weinberg equilibrium
ICD: International Classification of Diseases
*IL17RA*: Interleukin 17 receptor A (gene)
LD: Linkage disequilibrium
MAF: Minor allele frequency
MIS: Michigan Imputation Server
MSigDB: Molecular Signatures Database
NAFLD: Nonalcoholic fatty liver disease
NAS: NAFLD Activity Score
NASH: Nonalcoholic steatohepatitis
*NCAN*: Neurocan (gene)
*NFIL3*: Nuclear factor interleukin 3 regulated (gene)
NLP: Natural language processing
*PARVB*: Parvin beta (gene)
PC: Principal component
PheWAS: Phenome-wide association study
*PNPLA3*: Patatin-like phospholipase domain–containing 3 (gene)
*PPARG*: Peroxisome proliferator-activated receptor gamma (gene)
QC: Quality control
RELI: Regulatory Element Locus Intersection
ROC: Receiver operating characteristic curve
*SAMM50*: SAMM50 sorting and assembly machinery component (gene)
*SDC1*: Syndecan 1 (gene)
*SPI1*: Spi-1 proto-oncogene (gene)
*TM6SF2*: Transmembrane 6 superfamily member 2 (gene)
*TRIB1*: Tribbles pseudokinase 1 (gene)
*XDH*: Xanthine dehydrogenase (gene)
*ZFP90*: ZFP90 zinc finger protein (gene)
