# Supplementary material for: GWAS and enrichment analyses of non-alcoholic fatty liver disease identify new trait-associated genes and pathways across eMERGE Network
Source: BMC Med. 2019 Jul 17;17:135. doi: 10.1186/s12916-019-1364-z (PMC6636057; doi:10.1186/s12916-019-1364-z)

**Additional file 2**

**GWAS and enrichment analyses of Non-alcoholic fatty liver disease identify new trait-associated genes and pathways across eMERGE network**

Bahram Namjou^1,2*^, Todd Lingren^2,3^, Yongbo Huang^1^, Sreeja Parameswaran^1^, Beth L. Cobb^1^, Ian B. Stanaway^4^, John J. Connolly^5^, Frank D. Mentch^5^, Barbara Benoit^6^, Xinnan Niu^7^, Wei-Qi Wei^7^ Robert J. Carroll^7^, Jennifer A. Pacheco^8^, Isaac T.W. Harley^9^, Senad Divanovic^9^, David S. Carrell^10^, Eric B. Larson^10^, David J. Carey^11^, Shefali Verma^12^, Marylyn D. Ritchie^12^, Ali G. Gharavi^13^, Shawn Murphy^14^, Marc S. Williams^15^, David R. Crosslin^4^, Gail P. Jarvik^16^, Iftikhar J. Kullo^17^, Hakon Hakonarson^5,18^, Rongling Li^19^, The eMERGE Network^19^, Stavra A. Xanthakos^20^, John B. Harley ^1,2,21^

**Imputation and genetic analysis**

The imputation process has been recently described (23). Briefly, the publicly available Michigan Imputation Server (MIS) was used for phasing and imputation process. The genotyping data currently consist of 83,717 consented eMERGE participants in 78 batches provided as PLINK binary files. In this pipeline each array batch was imputed independently using minimac3 program as an imputation statistical model and Haplotype Reference Consortium HRC (version 1.1) as reference (24). All coordinates were in genome build 37. The quality of missing genotype imputation in the 78 genotype array batches was assessed using the r-square imputation quality metric as described in minimac3. This value (0-1) is an estimate of the squared correlation between the unobserved genotypes and the imputed genotypes. The finished MIS imputation data were provided by chromosome in VCF format with an underlying allele-dose model and the accompanying *.info files contain the r-squared quality correlation for each variant. After applying a conservative threshold of r2≥0.7, MAF of >1% and HWE p>0.0001, 7,263,501 autosomal variants were used for our study. Of note, the frequency bins 0.01 to 0.00001 represent the bulk (27,119,930 variants) of the imputed genotype spectrum (23). Autosomal markers with a MAF >0.05 after Linkage Disequilibrium (LD) pruning were used to create correlation matrix for Principal Component (PC) analysis using PLINK2 (25,26). PC ancestry analysis for the total 83717 eMERGE collection showed concordance with self-reported race without evidence of batch effects. PC1 explained 88.3% of the imputed genotype variance and represented the African to European ancestry while PC2 showed 6.1% of the genotype variance representing the Asian to European ancestry (23). Next, from this collection of 83717, a total of 9677 unrelated European ancestry participants (1106 cases and 8571 controls) with phenotypic information were selected to be evaluated for this study. Similar to above, after LD pruning on markers with a MAF >0.05 using PLINK2, a separate PC analysis was performed on this target population, the variance explained by each PC was calculated and the first three PCs were selected upon the examination of the scree plot that was used to account for population stratification (Figure S1). In addition to adjusting for the first 3 PCs, sites of genotyping (10 different medical centers), age, sex and body mass index were also included in the regression model for a total of 16 covariates.

**Post-GWAS analyses, and data visualization:** In order to further annotate, prioritize, and interpret GWAS results based on functional mapping, we used FUMA (Functional Mapping and Annotation of Genome-Wide Association Studies) platform with two available modules of SNP2GENE that initially takes GWAS summary statistics as an input, and provides extensive functional annotation for all SNPs in areas identified by lead SNPs and GENE2FUNCTION module that annotates genes in biological context for pathway enrichment analyses (33). FUMA incorporates 18 biological data repositories and tools to process GWAS summary statistics and provide a variety of annotations (33). SNP annotations included ANNOVAR, CADD (Combined Annotation-Dependent Depletion) score, effects on gene expression eQTLs in various tissue types using Genotype-Tissue Expression, GTEx-version 7 (34), RegulomeDB score and chromatin interactions. Additionally, we used Haploreg (V4) which includes four different options for defining enhancers using Roadmap Epigenomics (35). The R-package haploR was implemented to query HaploReg with batch of selected SNPs (36). In pathway enrichment analyses, FUMA uses hypergeometric test with a false discovery rate (FDR) of 0.05 for multiple hypothesis testing. Gene sets available in the Molecular Signatures Database (MSigDB) that are divided into 8 major collections (C1-C8) were primarily used for pathway-based analyses. Gene-based analyses was also performed using MAGMA both as part of FUMA platform and separately, to quantify the degree of association of genes by combining SNP p-values accounting for LD, gene size and gene density (37). We used a window of 20 kb upstream and downstream of a gene to define a gene. SNP to gene annotation of 7,263,501, results in 18375 protein-coding genes that used for significant threshold (0.05/18375=2.72 x 10^-6^) of MAGMA gene-based approach. Gene-property analyses to test relationships between tissue specific gene expression profiles and disease-gene associations were also performed using MAGMA (37). The goal of this analysis was to determine if tissue-specific differential expression levels were predictive of the association of a gene with NAFLD. Under FUMA module, the gene expression values of GTEx V7, have been normalized and used as a reference. Tissue expression analysis was performed for 30 general tissue types and 53 specific tissue types separately. Bonferroni correction was then used for multiple testing.

TF-enrichment analyses were performed using RELI (Regulatory Element Locus Intersection), a new in-house algorithm developed by our group (38). This enrichment algorithm is an extension of previous methods such as XGR (39). RELI estimates the overlap between an input set of genomic summary statistics, search for potential shared regulatory mechanisms acting across phenotype-associated loci and calculates the statistical intersection of the resulting loci with every dataset in our warehouse collection that currently consist of 1,544 ChIP-seq experiments, 344 TFs and 221 cell lines (38).The expected intersection distributions are Gaussian, and can hence be used to calculate Z-scores and P-values. The final reported P-values are Bonferroni corrected for the number of datasets tested. Finally, to graphically display results, LocusZoom and Golden Helix programs were used (40, 41).

**Supplementary Figure legend:**

Figure S1: The scree plot of proportion of variance (y-axis) explained by PC (1-10) (x-axis) and the selected threshold of factor retention (the elbow of scree plot) for 9677 European population.

**Figure S1:**


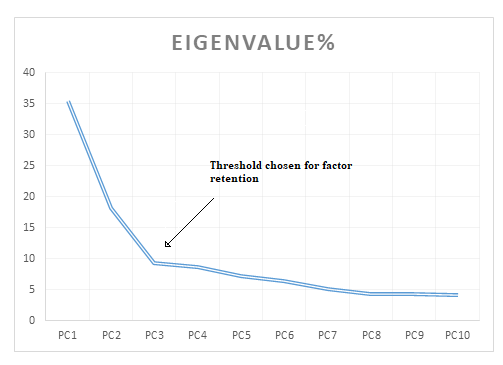

Supplement: Supplementary file 2 — Additional methodology. (DOC 53 kb) [file 12916_2019_1364_MOESM2_ESM.doc]
